# Metabolic Rate and Climatic Fluctuations Shape Continental Wide Pattern of Genetic Divergence and Biodiversity in Fishes

**DOI:** 10.1371/journal.pone.0070296

**Published:** 2013-07-29

**Authors:** Julien April, Robert H. Hanner, Richard L. Mayden, Louis Bernatchez

**Affiliations:** 1 Institut de Biologie Intégrative et des Systèmes (IBIS), Université Laval, Québec, Canada; 2 Ministère du Développement durable, de l’Environnement, de la Faune et des Parcs, Québec, Québec, Canada; 3 Biodiversity Institute of Ontario, University of Guelph, Guelph, Ontario, Canada; 4 Department of Biology, Saint Louis University, Saint Louis, Missouri, United States of America; Auburn University, United States of America

## Abstract

Taxonomically exhaustive and continent wide patterns of genetic divergence within and between species have rarely been described and the underlying evolutionary causes shaping biodiversity distribution remain contentious. Here, we show that geographic patterns of intraspecific and interspecific genetic divergence among nearly all of the North American freshwater fish species (>750 species) support a dual role involving both the late Pliocene-Pleistocene climatic fluctuations and metabolic rate in determining latitudinal gradients of genetic divergence and very likely influencing speciation rates. Results indicate that the recurrent glacial cycles caused global reduction in intraspecific diversity, interspecific genetic divergence, and species richness at higher latitudes. At the opposite, longer geographic isolation, higher metabolic rate increasing substitution rate and possibly the rapid accumulation of genetic incompatibilities, led to an increasing biodiversity towards lower latitudes. This indicates that both intrinsic and extrinsic factors similarly affect micro and macro evolutionary processes shaping global patterns of biodiversity distribution. These results also indicate that factors favouring allopatric speciation are the main drivers underlying the diversification of North American freshwater fishes.

## Introduction

Levels of genetic divergence and mutation rates vary between and among species as well as between geographic regions [1]–[4]. Identifying factors responsible for such variation is of fundamental interest because it might allow understanding general patterns of biodiversity distribution such as the latitudinal gradient of diversity, which ranks among the most striking and oldest recognized pattern in ecology [5]–[7]. It may even allow calibrating the molecular clock to better account for mutation rates variation [8]. Yet, despite much interest on the topic, there is an astonishing lack of consensus about the underlying evolutionary factor regulating biodiversity distribution and diversification.

Many factors have been hypothesised to influence global patterns of biodiversity distribution among taxonomic groups and geographic regions. A first general hypothesis suggests that climatic fluctuation of late Pliocene - Pleistocene glacial cycles has played a major role in shaping a latitudinal biodiversity gradient [5], [7], [9]. For instance, glacial cycles have certainly been the most dramatic events occurring during the lifespan of extant species [10], [11]. Thus, extinction rate, correlated with the severity of glaciations, may have been high near the poles and gradually lower towards the equator. Furthermore, levels of genetic divergence and eventually allopatric speciation near the tropics could be enhanced by the longer persistence of isolated populations.

A second general hypothesis suggests that metabolic rate influence the pace of evolution in acting on mutation rate [1], [2], [3], [8], [12]. In particular, higher metabolic activity is associated with higher oxygen consumption and release of free radicals, which may in turn induce higher mutation rates and rapid genetic divergence. It has been demonstrated that mass specific metabolic rate decrease with body size and increase with temperature [13]. Therefore, the generally higher temperature found at lower latitude might increase the tempo of molecular evolution through metabolic rates near the tropics compare to polar region.

A full understanding of the relative role of climatic fluctuation and metabolic rate in shaping global patterns of diversity requires not only investigating geographic patterns of species richness but also those of genetic divergence at both the intraspecific and interspecific levels [7], [14]. Thus, congruent patterns of variation in genetic divergence within species and genetic divergence between sister species, along abundantly documented geographic variation in species richness, would make a strong case for a causal link between these factors and potential for diversification and speciation. Yet, surprisingly few studies aimed at identifying factors influencing recent speciation events and even fewer have investigated simultaneously genetic patterns of both the intraspecific and interspecific levels (but see [14], [15]). Moreover, only a handful of studies investigated jointly the role of both intrinsic (e.g. metabolic rate) and extrinsic (e.g. glacial cycles) factors in shaping large scale patterns of biodiversity [7]. Even more rarely have those questions been addressed using data from the whole distribution range of an entire taxonomic community. Finally, conclusions of previous studies addressing those issues may have been affected by taxonomic biases associated with either economic or scientific interest [16].

North American freshwater fish fauna represent an exceptional model to investigate the determinants of global pattern of biodiversity. First, it is species rich with 903 formally recognised species in the U.S. and Canada, and yet most species are endemic to the continent [17], [18]. Given the inherent structure of their habitats, freshwater fishes have limited dispersal opportunities. As such, they represent one of the most highly genetically structured taxonomic groups [19], [20]. Therefore, migration out of the tropics (see [21]) may be of negligible importance in freshwater fishes compare to mobile organisms such as marine bivalves, birds or large mammals, and this is especially true at large scale [22]. This entire fauna has been studied for decades in both Canada and the United-States [23]–[25]. Therefore, there is no obvious reason why proportion of putatively undescribed species would show an important geographical bias, at least compared to studies involving tropical regions where most undescribed species are thought to occur [16], [26], [27]. The distribution of North American freshwater fishes is also characterised by a pronounced latitudinal gradient of species richness which decreases from 500 species at the lowest latitude to one at the highest latitudes [18], and it has been hypothesised that this pattern reflects a latitudinal variation in speciation/extinction rates [7]. A previous study based on the analysis of a limited (n = 42) number of species and using various published mitochondrial DNA data sets also revealed an overall reduction of intraspecific genetic diversity with latitude [15]. Another study based on 54 North American freshwater fish has found evidence that metabolic rates might influence substitution rates [3]. Finally, April et al. [20] developed a mitochondrial DNA sequence (cytochrome c oxidase subunit 1, mean = 650 bp) dataset based on 5,674 individuals from 752 species, representing the almost entire North American freshwater fish fauna. The congruence between mtDNA divergence and delineation of either taxonomically recognised species (90%) or geographically isolated populations within species (88%) was remarkable, which confirmed that in freshwater fishes at least, mtDNA is an excellent index of both the extent of geographic isolation and species divergence.

In this study, we test the two general hypotheses that temporal habitat stability associated with the late Pliocene - Pleistocene glacial cycles, as well as metabolic rate associated with thermal regime and size, have dually contributed in shaping global patterns of intraspecific and interspecific genetic divergence. More specifically, we predict 1.a) a negative relationship between genetic divergence and latitude, as well as 1.b) a breakpoint in this relationship located at the known southern limits of Wisconsinan glaciers (46°), if glacial cycles have importantly influenced current patterns of genetic divergence. Under the hypothesis that metabolic rates largely influence genetic divergence and mutation rates, we predict 2.a) a positive relationship between genetic divergence and metabolic rate, 2.b) that the explanatory power of metabolic rate should be higher than the ones of body size and temperature, as well as 2.c) a tendency for mutation rates to increase with metabolic rate. To test those hypotheses, we measured the extent of mtDNA phylogroup divergence within species and the extent of genetic divergence between closely related species from the majority of North American freshwater fish species. We then use generalized linear mixed models to assess the relationship of genetic divergence with latitude, metabolic rates, body size and temperature. We verify the effect of metabolic rate on mutation rate by using ratio of branch length at node between sister species.

## Materials and Methods

### Data Acquisition

We first obtained two datasets, one at the intraspecific level and the other at the interspecific level, including only native North American species that spend their entire life in freshwater, thus excluding those that are obligatory diadromous or mainly occurring in saltwater. These two datasets were obtained from an initial dataset including 5674 specimens from 752 species [20] (Figure S1). All cytochrome c oxidase 1 sequences were over 500 bp long (mean = 648 bp). For the intraspecific dataset, we estimated genetic divergence between intraspecific phylogroups by measuring the mean genetic distance between individuals of the same species. In 88% of the cases, intraspecific divergence was observed between individuals from different sampling sites rather than from the same sampling sites [20]. Therefore, those phylogroups defined at the intraspecific level most likely identify intraspecific evolutionary lineages that have been geographically isolated for variable period of times [20], [28]. Because the level of phylogroup divergence was correlated with the number of states and provinces sampled locations (r = 0.33, p-value<0.0001), which is indicative of the effects of both the number of sampling sites and the geographic distance between sites, we computed corrected genetic divergence values. We standardised the phylogroup divergence by dividing the distance value by the total number of sampled states/provinces (mean = 2.3). This phylogroup dataset includes 550 species (Text S1) spanning seven orders for which an average of 8.8 individuals from multiple geographic locations (mean = 3.8 sites/species) were sequenced. At the interspecific level, we computed genetic divergence between nearest neighbour for each species (sensu [29]). This represents the distance between the focal species and its closest relative. We did not included species for which the sister species occurred only on another continent and species that are the only representative of their family in the dataset. We also excluded species that were part of a species complex and sharing haplotypes with another species, representing 10% of the total number of species analysed by April et al. [20]. This is because this phenomenon can be explained by different scenarios with different evolutionary meanings (e.g. hybridization between old lineages and young lineages sharing ancestral polymorphisms). However, we verified if this choice affect our results by also conducting the analyses with a dataset including those species and obtained similar results (Table S1).The nearest neighbour species dataset includes 510 species (Text S2). Mean sequence divergence estimates, between phylogroups within species or between nearest neighbour species, were calculated using the Kimura 2-parameters model [30] on the program BOLD [31]. Phylogenetic tree and sampling information for all species is available on the BOLD website in the project “North American freshwater fish” (www.boldsystems.org).

For each species included in the analysis, we first used extent literature [32]–[34] to estimate the midpoint latitude of the distribution range as a relative proxy for long term latitudinal distribution of a given species. We also estimated the relative mass specific metabolic rate using the formula of Gillooly et al. [13]: B = b_o_M −1/4 e−E/kT, where B is the metabolic rate, b_o_ is a normalization coefficient independent of mass and temperature, M is body mass, e−E/kT is the Boltzmann factor, which underlies the temperature dependence of metabolic rate (E is the activation energy of the rate-limiting biochemical reactions of metabolism [0.65 eV], k is Boltzmann’s constant [8.62×10−5 eV K−1], and T is absolute temperature [K]). To estimate body mass, we first obtained maximum total length from literature for each species [33], [34]. We then transformed maximum total length to weight following the equation of Carlander [35] (see also [3]). Since fishes are poikilotherms, body temperature was approximated using annual mean ambient temperature in Kelvin degree (see also [3]). This was measured by first estimating the center of the distribution range of each species (both latitude and longitude) using the literature [32], [33]. We then used data from the American National Oceanic and Atmospheric Administration and from Environment Canada to obtain the mean annual ambient temperature at those locations. Because temperature estimates incorporates climatic and elevation information, this variable remain relatively independent of midpoint latitude (r^2^ = 0.48, Table S2).

### Statistical Analyses

We tested the relationship between the genetic divergence parameters and the hypothesised explanatory variables using generalized linear mixed models (GLMM). Such models are particularly well suited for dealing accurately with non-normal data and random variables [36]. The relationship of both phylogroups and nearest neighbour species genetic divergence with a) midpoint latitude, b) mass specific metabolic rate, c) body size and d) temperature was modeled using a binomial distribution. We used the taxonomic rank order as a random variable to control for phylogenetic bias through a hierarchic model and to estimate its effect using a log-likelihood ratio test. To get sufficient statistical power, we only included in the analyses the orders represented by at least 5 species in our datasets. At the end, both phylogroups and nearest neighbour species datasets included the same 6 orders in addition to one different order in each dataset, for a total of 7 orders. Semionotiformes were not included in the phylogroup dataset because multiple individuals have been analysed for less than 5 species. Esociformes was not included in the nearest neighbour species datasets because less than 5 species have a sister-species occurring in North America. For all generalized linear mixed models, the goodness of fit of the models was compared using an information criterion (AIC [37]) and p-values were obtained using z-values. All the generalized linear mixed models analyses were conducted in R using the package lme4 (R Development Core Team 2008).

### Additional Tests

We performed an additional test in order to further assess the association between genetic divergence and the late Pliocene–Pleistocene glaciations events. We predicted a breakpoint in the relationship between sequence divergences and midpoint latitude located at the known southern limits of Wisconsinan glaciers (46°), if glaciations had a significant impact on geographical patterns of genetic divergence. We tested this prediction using piecewise generalized linear mixed models, involving or not a breakpoint at 46° of latitude, between both phylogroup and nearest neighbour species sequence divergences and midpoint latitude. Lower AIC values [37] for the models implying the breakpoint would suggest an important effect of glaciers on genetic patterns of divergence. As for the other generalized linear mixed models analyses, piecewise regressions were conducted in R (R Development Core Team 2008) using order as a random variable.

We also performed an additional test in order to further investigate the effect of mass specific metabolic rate on patterns of sequence divergence and directly tested if mutation rates increase with mass specific metabolic rate. Therefore, we measured relative mutation rate by using the ratio of branch length at node between sister species under the following rationale. For any given pair of sister species, the sister is each other’s closest relative and thus form a monophyletic group. Their time of divergence from the closest common ancestor is thus the same and differences in the number of mutations between the node and the tips of two sister species is theoretically predicted to reflect differences in mutation rates [3], [4], [38]. Under the hypothesis of a causal relationship between mass specific metabolic rate and mutation rate, we predicted a positive relationship between both parameters. For each taxon, we first used Mega 5 [39] to find the best substitution model based on Bayesian Information Criterion. With the same software [39], we then computed maximum likelihood phylogenetic trees using the selected substitution models [40]. Following a heuristic search, we kept the tree with the highest log likelihood. We obtained a total of 123 sister species pairs (total = 246 species; Text S3). This sister-species pair dataset represent a fraction of the nearest neighbour species dataset because the selection criteria for sister-species (pair of species that are each other’s closest relative and forming a monophyletic group at terminal branches) is more restrictive than the criteria for nearest neighbour species (a species that is the closest living species of another species). For all recovered sister species pairs, we measured the branch length from the tips to the nodes separating the sister pairs [4]. We then conducted a non-parametric 2-tailed sign test to determine whether for each pair; the species with higher mass specific metabolic rates also tends to accumulate more mutations relative to the other species. Since this analysis can take into account the sign of the relationship but not the amplitude of the variation, we also conducted a parametric linear regression analyses. For each sister species pair, we calculated the metabolic rate contrast as the logarithm of the mass specific metabolic rate of the first species divided by the mass specific metabolic rate of the second species [38]. We also calculated the contrast in mutation rate as the natural logarithm of the branch length of the first species divided by the branch length of the second species of the sister pair. Those two variables were used in a regression that was forced through the origin [4], [38]. Those statistical analyses were conducted in R (R Development Core Team 2008).

We also verified if there is a correlation between genetic divergence at both mitochondrial and nuclear DNA levels using published data [41]. From a North American cyprinids phylogeny [41], which is the largest family of North American freshwater fishes, we analysed nearest neighbour species divergence for 36 species from 8 different genera for which both mitochondrial DNA (CytB) and nuclear DNA (Rag1) was sequenced (Text S4). The relationship between the two measurements of genetic divergence was assessed using a Pearson’s correlation conducted in R (R Development Core Team 2008).

## Results

**Figure 1:**
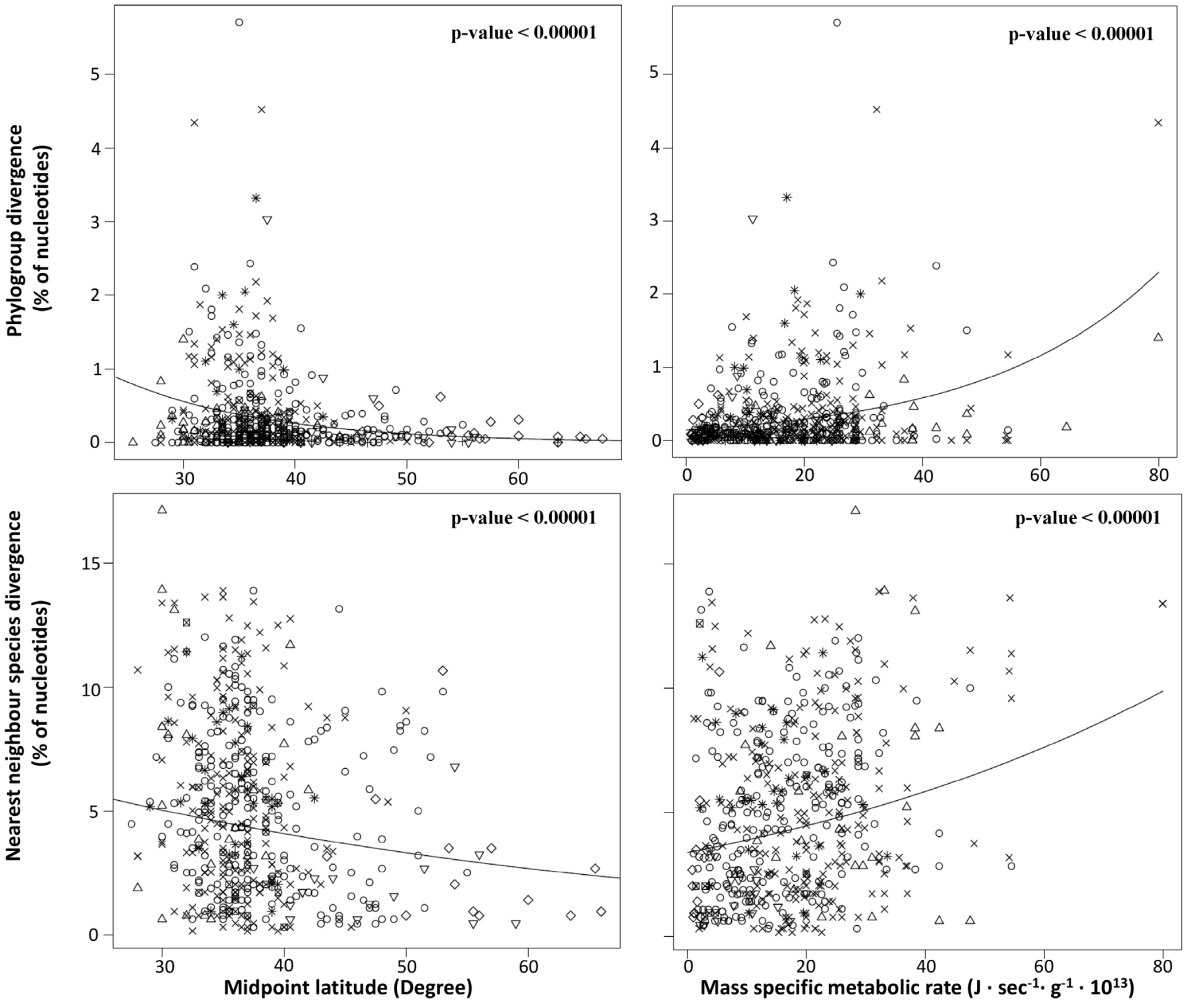
Plots of genetic divergence against midpoint latitude and mass specific metabolic rate using raw data. Each sign represents a different order (○ = Cypriniformes, × = Perciformes, Δ = Cyprinodontiformes, ∇ = Scorpeiniformes, ⊠ = Semionotiformes, * = Siluriformes, ◊ = Salmoniformes, ⊞ = Esociformes). Fitted generalized linear mixed models show a significant relationship between variables in all comparisons (see Table 1).

**Figure 2:**
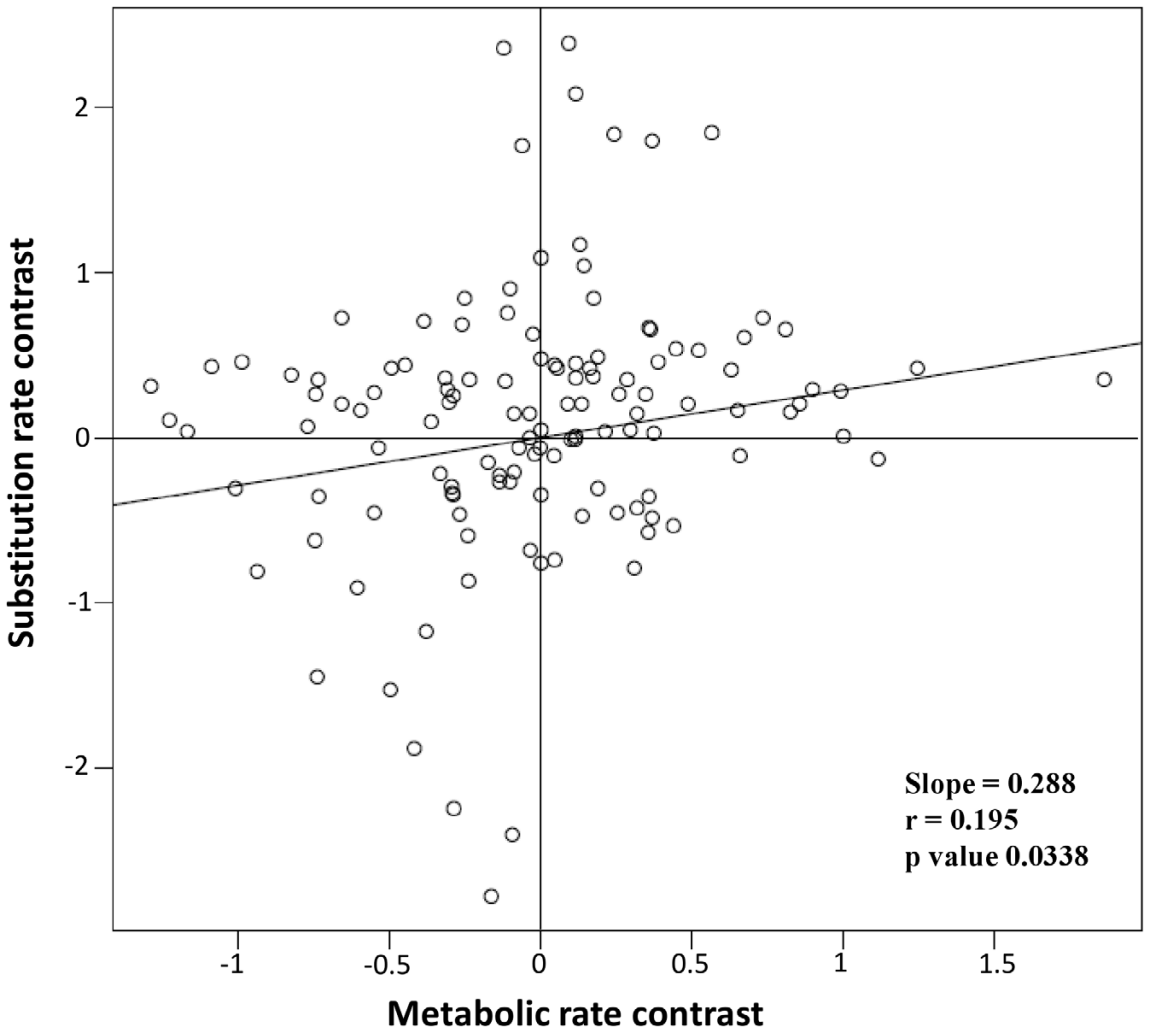
Plots of phylogenetically independent sister pair comparisons of substitution rate against mass specific metabolic rate. The X values correspond to the natural logarithm of the mass specific metabolic rate of the first species divided by the mass specific metabolic rate of the second species. The Y values correspond to the natural logarithm of the branch length of the first species divided by the branch length of the second species. Regression line was forced through the origin.

Our results show a highly significant effect of both midpoint latitude and mass specific metabolic rate on the extent of phylogroup genetic divergence whereby phylogroup sequence divergence decreases with midpoint latitude and increases with mass specific metabolic rate (Table 1, Figure 1). The best model, based on the lowest Akaike information criterion value (AIC [37]), was obtained for the variable mass specific metabolic rate. At the interspecific level, there was also a highly significant effect of both midpoint latitude and mass specific metabolic rate on the extent of divergence between nearest neighbour species, and the effect of these factors were in the same direction than observed at the intraspecific level (Table 1, Figure 2). Here again, the best model (lowest AIC) was obtained for the variable mass specific metabolic rate. The variables “body size” and “temperature” had also an effect on genetic divergence, but was always weaker than the effect of mass specific metabolic rate and non-significant in some cases (Table 1). However, the sign of the relationships followed the prediction that both intraspecific and interspecific divergence should increase with temperature and decreased with body size.

**Table 1:** Results of generalized linear mixed models.

		Phylogroup divergence				
Model/Hypothesis	Parameter	Estimate	Std. Error	z-value	p-value	AIC
**Climatic fluctuation**	Midpoint latitude	−0.0775	0.0074	−10.50	<2e-16 ***	2037
	Intercept	−2.8564	0.3587	−7.96	1.66e-15 ***	
Breakpoint at glacier margin	Latitude<46^o^	−0.0809	0.0092	−8.82	<2e-16 ***	2038
	Latitude >46^o^	−0.0782	0.0074	−10.53	<2e-16 ***	
	Intercept	−2.7479	0.3925	−7.00	2.55e-12 ***	
**Metabolic rate**	Mass specific metabolic rate	0.0348	0.0022	15.64	<2e-16 ***	1957
	Intercept	−6.5360	0.2332	−28.02	<2e-16 ***	
Generation time	Body size	−0.0218	0.0024	−9.01	<2e-16 ***	2037
	Intercept	−5.4307	0.1795	−30.25	<2e-16 ***	
Temperature	Temperature	0.0930	0.0087	10.75	<2e-16 ***	2037
	Intercept	−7.1448	0.2204	−32.41	<2e-16 ***	
**Best complex model**	Midpoint latitude	−0.0373	0.0087	−4.30	1.74e-05 ***	1918
	Body size	−0.0102	0.0024	−4.26	2.05e-05 ***	
	Mass specific metabolic rate	0.0221	0.0032	6.85	7.34e-12 ***	
	Intercept	−4.5872	0.4565	−10.05	<2e-16 ***	
		**Nearest neighbour species divergence**				
**Model/Hypothesis**	**Parameter**	**Estimate**	**Std. Error**	**z-value**	**p-value**	**AIC**
**Climatic fluctuation**	Midpoint latitude	−0.0219	0.0019	−11.63	<2e-16 ***	8040
	Intercept	−2.2811	0.1385	−16.47	<2e-16 ***	
Breakpoint at glacier margin	Latitude<46^o^	−0.0322	0.0025	−12.92	<2e-16 ***	8002
	Latitude >46^o^	−0.0261	0.0019	−13.20	<2e-16 ***	
	Intercept	−1.9383	0.1527	−12.69	<2e-16 ***	
**Metabolic rate**	Mass specific metabolic rate	0.0143	0.0007	20.40	<2e-16 ***	7785
	Intercept	−3.3533	0.1271	−26.37	<2e-16 ***	
Generation time	Body size	−0.0006	0.0004	−1.63	0.103	8175
	Intercept	−3.1296	0.1528	−20.48	<2e-16 ***	
Temperature	Temperature	0.0283	0.0023	12.57	<2e-16 ***	8017
	Intercept	−3.5303	0.1313	−26.89	<2e-16 ***	
**Best complex model**	Midpoint latitude	−0.0104	0.0032	−3.17	0.00154 **	7701
	Body size	0.0038	0.0004	9.36	<2e-16 ***	
	Temperature	−0.0183	0.0045	−4.06	4.86e-05 ***	
	Mass specific metabolic rate	0.0193	0.0011	18.37	<2e-16 ***	
	Intercept	−2.9416	0.2221	−13.24	<2e-16 ***	

Tests were conducted at the intraspecific level using phylogroups (upper table) and at the interspecific level using nearest neighbour species (lower table) mtDNA sequence divergence data. For the model testing the presence of a breakpoint at glacier margin, we conducted a piece-wise regression involving a break at 46^o^ of latitude which corresponds to the maximal extent of Pleistocene glaciations events.

Results of the generalized linear mixed model analysis using different combination of explanatory variables support the results of the generalized linear mixed model conducted using both variables independently. We only present the model having the lowest AIC value, considered to be the best model, in Table 1. Based on z-values, mass specific metabolic rate always appeared to be the factor with the highest explanatory power influencing both phylogroup and nearest neighbour species genetic divergence. This relationship remains positive and significant in all cases. The slope of the relationship between midpoint latitude and both phylogroup and nearest neighbour species divergence also remains negative and significant. At the phylogroup level, the variable body size was also included in the best model which remained significant and negative. At the neighbour species level, the best model included also body size and temperature which had a significant effect. However, contrary to the prediction and to the analyses conducted independently for each variable, body size appears to have a positive effect on genetic divergence while the effect of temperature appears negative.

The random effect order had a highly significant effect in all models (p-values<0.000001), meaning that some orders were characterized by higher levels of intraspecific or interspecies divergence than others. For example, levels of phylogroup divergence are the highest in Siluriformes (mean = 1.37 percent of nucleotide divergence, range: 0 to 6.4) and particularly low in Esociformes (mean = 0.12 percent of nucleotide divergence, range: 0 to 0.62). For nearest neighbour species divergence, the extremes were observed in Siluriformes (mean = 6.07 percent of nucleotide divergence, range: 0.93 to 13.89) and Scorpaeniformes (mean = 1.92 percent of nucleotide divergence, range: 0.46 to 6.78).

We observed that the piecewise regression involving a breakpoint at glacier margin (46°) had a better fit (lower AIC) than the simple midpoint latitude model in explaining patterns of genetic divergence between nearest neighbour species, but not for phylogroup divergence within species (Table 1). However, for both phylogroup and nearest neighbour species, the predicted effect of midpoint latitude on the extent of sequence divergence was more pronounced to the south of the glacier margin (Latitude<46°) than north, as exemplified by differences in estimates and z-values.

We found a significant relationship between mutation rate and mass specific metabolic rate. Results of non parametric 2-tailed sign test (74 pairs showing predicted trend Vs 49 pairs not showing predicted trend, p-value = 0.030) and parametric linear regression (slope = 0.091, r = 0.195, p-value = 0.028) support the hypothesis that species with higher mass specific metabolic rate had significantly higher mutation rates. We found no relationship between mutation rate and body size (slope = 0.0005, r = −0.003, p-value = 0.973). Based on our analyses of a published North American cyprinid phylogeny that used both mitochondrial and nuclear DNA data sequences [41], we found that levels of mtDNA divergence is a reliable indicator of the level of divergence observed at nucDNA. Indeed, there was a highly significant correlation between the two measurements of genetic divergence (r = 0.643, p-value = 0.004).

## Discussion

### Glacial Cycles of the Pleistocene and Late Pliocene

Our results globally support the hypothesis that late Pliocene – Pleistocene climatic fluctuations that caused glacial cycles had a generalised effect on both the patterns of intraspecific (phylogroup) and interspecific (nearest neighbour species) genetic divergence over the whole North American freshwater fish fauna. We observed a significant and negative relationship between latitude and genetic divergence as well as a breakpoint in the relationship that corresponds to the southern extends of the Wisconsinan ice sheet in the case of interspecific divergence (Table 1). Even using a wide range of possible mitochondrial DNA mutation rate (1 to 4 percent sequence divergence per million years; [42], genetic divergence observed for phylogroup (mean = 0.78% of nucleotide divergence; ∼195 000 to 780 000 years) and nearest neighbour species (mean = 5.03% of nucleotide divergence; ∼1.3 to 5 million years) suggest that most diversification events occurred during the glacial cycles of the Pleistocene and late Pliocene. The observed reduction of intraspecific and interspecific genetic divergence with latitudes had been previously reported based on a meta-analysis comprising heterogeneous data sets from 42 fish species [15]. Our study, based on the analyses of over 500 species and using standardized genetic data, thus generalises these latitudinal gradients to the whole North American freshwater fish fauna. Such increase of intraspecific and interspecific genetic divergence toward lower latitude has further been documented in birds and mammals [14]. Climatic variation associated with glaciations most likely created a latitudinal variation in extinction and speciation rate caused by variable extant of habitat stability. Thus, reduction in abundance and extinction were almost certainly more prevalent at northern latitude where habitats were recurrently devastated by as many as 16 glacial advances during the Pleistocene [43]. In contrast, although habitat shifts also certainly occurred further south, habitat and associated fish fauna were more likely to persist for longer periods of time in separated water drainages, as suggested for fish and other vertebrates [10], [15], [44], [45]. Thus, assuming that the extant of genetic divergence correlates with time [46], more recent geographic isolation should translate into a decrease of phylogroup and nearest neighbour species sequence divergence with midpoint latitude, as globally observed here [11], [15]. In corollary, increased phylogroup and nearest neighbour species divergence is expected at more southern latitudes.

The generally lower level of sequence divergence between nearest neighbour species at higher latitudes may suggest younger burst of ecological speciation influenced by climatic fluctuation [14]. Theoretical and empirical studies indicate that particularly rapid speciation can be achieved through ecological selection in sympatry [47]–[50]. This is also supported by theory predicting that high ecological opportunity, such as colonization of resource-rich environments free of competitors, will promote rapid phenotypic evolution leading to speciation [51]. Newly aquatic habitats that were created by glacier retreats are considered to have provided such opportunities that may have enhanced species diversification implying a sympatric phase [52]. Indeed, many of the best documented cases of recent ecological speciation following the last (Wisconsinan) glaciations involve freshwater fishes found at high latitudes (*Coregonus* sp. [47], *Gasterosteus* sp. [48], *Oncorhynchus* sp. [53]). As a whole, however, such very recent cases of ecological and at least partially sympatric speciation are often geographically localized and might be relatively rare compared to cases of allopatric speciation[54]–[57]. For instance, clear cases of ecological speciation in the three-spined stickleback (*Gasterosteus* sp.) in North America are limited to a few lakes of the north-western Pacific coast whereas the species is ubiquitous to coastal areas all over North America [54], [58]. Young burst of ecological speciation associated with postglacial lakes certainly did occur in northern latitudes [14], [54]. However, the restricted level of genetic divergence (Table 1) and species diversity [18] accumulated in northern latitudes indicate that the repeated glacier advances typically erased the newly evolved diversity either directly or by promoting extinction through hybridisation [59]–[62] in the disturbed environment that represented post-glacial transitional watersheds.

The potential for genetic divergence and allopatric speciation might have been greater at southern latitudes because phylogroups and species occurring in these temporally more stable environments could survive long periods of time in geographic isolation (that is lower extinction rate associated with habitat loss), and thus accumulate more pronounced genetic differences. Here, we reiterate that the majority of analyzed intraspecific phylogroups are geographically partitioned (88%) [20]. Thus, more pronounced phylogroup divergence towards the south likely indicates longer geographic isolation. Increased probability of genomic incompatibility with increased time of divergence has been demonstrated in many animal taxa (insects, amphibians, birds; reviewed in [55], [63], [64]). This predicts that longer isolation may also translate into increased probability to achieve reproductive isolation and speciation in southern latitudes. Indeed, positive relationship between genetic divergence and reproductive isolation has also been demonstrated in different families of North American freshwater fishes [28], [65], [66]. Another line of evidence suggesting that longer geographic isolation may have increased probability of allopatric speciation towards southern latitudes comes from the latitudinal distribution of deeply divergent phylogroups. Indeed, intraspecific phylogroups diverging by more than 2% at mitochondrial DNA could represent incipient species or young undescribed species [20], [28]. This is further supported by several studies showing that fish with such level of genetic divergence, or even lower, commonly remain reproductively isolated in sympatry [67]–[69]. Here, we found that the proportion of species with deep divergent phylogroups (>2%) decrease with latitude (R^2^ = 0.475, p-value = 0.0296), indicating that more lineages might be near the final stage of allopatric speciation in southern latitudes relative to northern.

### Metabolic Rates

Our results also suggest that metabolic rates significantly impact the continental wide pattern of intraspecific and interspecific sequence divergence of the North American freshwater fish fauna. This was supported by the positive and significant relationship between metabolic rates and genetic divergence, by a better model fit for this factor compared to midpoint latitude, body size and temperature (Table 1 and Table S1), as well as by the significant correlation between mutation rate and mass specific metabolic rate (Figure 2). As such, our results support the general hypothesis that mutation and divergence rates are mediated by the mutagenic effect of oxygen radicals produced with aerobic respiration and by the increased rate of DNA replacement and synthesis in organism with higher metabolic rates [1], [2]. This result is in agreement with a previous study based on 54 fish species that also showed a relationship between mitochondrial DNA mutation rate and mass specific metabolic rate [3].

Higher mutation rates may induce faster divergence and speciation rates [2], [12], [49], [70], [71]. Indeed, since most species are geographically structured, different mutations are expected to appear in different populations. According to the neutral theory of molecular evolution [70], the fixation rate of those new mutations is equal to the mutation rate and independent of population size. Under the classic Dobzhansky-Muller model [73], [74], as well as under other multilocus models of speciation through genetic incompatibilities, the average waiting time to speciation represent the time that it takes to get from the ancestral state to the states of fixation for the incompatibles alleles [49]. In allopatric speciation models based on mutation and random genetic drift, which represents a general null model of speciation, the average waiting time until fixation of a neutral allele is approximately the reciprocal of the mutation rate [49]. Thus, theory predicts that mutation rate is directly correlated with speciation rates. Models of parapatric and sympatric speciation also suggest that speciation rate should increase with mutation rates [49]. Therefore, the faster mutation rate observed for fish with higher metabolic rates, and generally located at southern latitudes, should allow those lineages to accumulate genetic differences and incompatibility faster than for northern taxa. Hence, for a similar period of allopatric isolation, southern phylogroups may be more likely to achieve a level of divergence preventing phylogroup mixture and reversibility (sensu Muller [75]) in secondary contact zone. This does not deny the role of extrinsic pre-zygotic isolation that can have an important role in speciation, but such barriers may be easier to reverse than intrinsic post-zygotic barrier that are likely to be permanent [55], [60], [61], [62], [75].

Although it often remains challenging to tease apart the effect of correlated characteristics, our results suggest that mass specific metabolic rate have a prime effect on genetic divergence and molecular evolution. Indeed, mass specific metabolic rate is correlated with the thermal environment, particularly so in poikilotherms. Thus, mass specific metabolic rate is also expected to covariate with latitude, which likely explains the similar patterns observed for both factors. However, the fact that the model considering mass specific metabolic rate had a better fit and explanatory power than the midpoint latitude and temperature models (Table 1 and Table S1) and that it correlated with mutation rate (Figure 2) suggests that it affected the observed patterns of intraspecific and interspecific divergence for additional causes differing from those inferred for latitude alone (temporal stability in populations structure and demography). Beside temperature, metabolic rate may also correlate with generation time and longevity that also vary with body size. For instance, generation time may be a direct consequence of metabolic rates [2], [13]. For fish, rigorous information directly concerning generation time and longevity are lacking for numerous species which prevented us to directly include those variables in our analyses. Nevertheless, the fact that we always observed a stronger effect of metabolic rates than for body size suggest that factors more directly correlated to body size, such as generation time, are less important in driving the observed metabolic rate.

### Latitudinal Gradient of Biodiversity

We thus propose that across the whole North American freshwater fish biota, genetic divergence and speciation rates has been particularly high in southern latitude because of more stable environments, allowing longer geographic isolation, coupled with a faster pace of molecular evolution causing more rapid accumulations of genetic differences and incompatibilities. This coupled, in conjunction with a lower probability of extinction relative to northern latitudes, may be largely responsible for the latitudinal gradient of species richness observed among North American freshwater fishes [18].

By simultaneously considering the dual roles of two very distinct mechanisms generating a spatial variation in intraspecific and interspecific genetic divergence, this study sheds new light on some longstanding questions in ecology and evolution. First, while some previous studies have suggested that current latitudinal gradient of diversity is primary the results of extrinsic factors [5], [14] whereas others advocated a primary role of intrinsic factors [2], [76], our results demonstrate that both factors are important and act jointly to create biodiversity latitudinal gradients (Table 1, Table S1). A second persistent debate concerns the link between microevolution and macroevolution. Indeed, doubts have been cast on the possibility that both processes could be governed by the same principles, in part because it is difficult to reconcile the apparent punctuated evolution observed at macro scale with the gradual process involve in microevolution [77]. As suggested by Hudson [78], differential patterns of genetic variation at both the intraspecific and interspecific levels would illustrate differential selective and/or demographic effects at the two levels. Here, results show that both level of biodiversity are influenced in a similar way by the same factors and as such, suggests that there might be continuity between microevolution and macroevolution, in accordance with the hypothesis that all levels of biodiversity must be influenced by origination and extinction rate [79]. Thirdly, while some controversy persists in describing southern biomes (e.g. tropics) as either museums or cradles of diversity [80], our results, along with other studies [7], [21], are indicative that there might well be both. Finally, while this study is noteworthy because it examines an entire fauna over an entire continent, the growth of DNA barcode records via the International Barcode of Life project (iBOL.org) will soon allow testing the generality of this finding in different scales, regions and taxonomic groups.

### Data Accessibility

Sequence data are available on GenBank (accession nos. EU522398–EU522464, EU523870–EU525162, HQ556931, HQ556937–HQ556979, HQ556989–HQ556990, HQ557037–HQ557038, HQ557067–HQ557069, HQ557071–HQ557076, HQ557086–HQ557089, HQ557095–HQ557097, HQ557114, HQ557121–HQ557132, HQ557136–HQ557222, HQ557262–HQ557272, HQ557285–HQ557286, HQ557301–HQ557365, HQ557375–HQ557395, HQ557397–HQ557464, HQ557467–HQ557471, HQ557475, HQ557489, HQ557495–HQ557497, HQ557524–HQ557555, HQ557720–HQ557733, HQ579002–HQ579067, HQ579071–HQ579136, HQ937011–HQ937054, HQ971430–HQ971434, and JN024710–JN028456). DNA sequences and sampling information for all species are available on the BOLD website in the project “North American freshwater fish” (www.boldsystems.org).

## Supporting Information

Figure S1 **Tree including all North American freshwater fish samples used in this study (5 674 specimens from 752 species).** This dataset is described in detail by April and collaborators (2011).
(DOCX)

Table S1 **Results of generalized linear mixed models using a dataset that includes species sharing haplotypes.** For the model testing the presence of a breakpoint at glacier margin, we conducted a piece-wise regression involving a break at 46° of latitude which corresponds to the maximal extent of Pleistocene glaciations events.
(DOCX)

Table S2 **Pearson correlations between explanatory variables.**
(DOCX)

Text S1 **List of species included in the intraspecific divergence analyses.**
(DOCX)

Text S2 **List of species included in the interspecific divergence analyses.**
(DOCX)

Text S3 **List of species included in the analyses testing the correlation between mass specific metabolic rate and mutation rate.**
(DOCX)

Text S4 **List of species included in the analyses testing the correlation between mtDNA divergence and nucDNA divergence Data from: Schönhuth S, Mayden RL (2010) Phylogenetic relationships in the genus** ***Cyprinella*** **(Actinopterygii: Cyprinidae) based on mitochondrial and nuclear gene sequences. Mol Phylo Evol 55∶77–98.**
(DOCX)
