# Supplementary material for: Metabolic Rate and Climatic Fluctuations Shape Continental Wide Pattern of Genetic Divergence and Biodiversity in Fishes
Source: PLoS One. 2013 Jul 29;8(7):e70296. doi: 10.1371/journal.pone.0070296 (PMC3726496; doi:10.1371/journal.pone.0070296)
Supplement: Figure S1 — Tree including all North American freshwater fish samples used in this study (5 674 specimens from 752 species). This dataset is described in detail by April and collaborators (2011). (DOCX) [file pone.0070296.s001.docx]

**Supplementary figure**

**Figure S1.** Tree including all North American freshwater fish samples used in this study (5 674 specimens from 752 species). This dataset is described in detail by April and collaborators (2011).
