# Supplementary material for: Metabolic Rate and Climatic Fluctuations Shape Continental Wide Pattern of Genetic Divergence and Biodiversity in Fishes
Source: PLoS One. 2013 Jul 29;8(7):e70296. doi: 10.1371/journal.pone.0070296 (PMC3726496; doi:10.1371/journal.pone.0070296)
Supplement: Table S1 — Results of generalized linear mixed models using a dataset that includes species sharing haplotypes. For the model testing the presence of a breakpoint at glacier margin, we conducted a piece-wise regression involving a break at 46° of latitude which corresponds to the maximal extent of Pleistocene glaciations events. (DOCX) [file pone.0070296.s002.docx]

**Supplementary Table**

**Table S1.** Results of generalized linear mixed models using a dataset that includes species sharing haplotypes. For the model testing the presence of a breakpoint at glacier margin, we conducted a piece-wise regression involving a break at 46^o^ of latitude which corresponds to the maximal extent of Pleistocene glaciations events.

|  |  | **Nearest neighbour species divergence** |  |  |  |  |
| --- | --- | --- | --- | --- | --- | --- |
|  |  | (including species sharing haplotypes) |  |  |  |  |
| **Model/Hypothesis** | **Parameter** | **Estimate** | **Std. Error** | **z-value** | **p-value** | **AIC** |
| **Climatic fluctuation** | Midpoint latitude | -0.0186 | 0.0016 | -11.33 | <2e-16 *** | 12995 |
|  | Intercept | -2.3373 | 0.2022 | -11.56 | <2e-16 *** |  |
| Breakpoint at glacier margin | Latitude < 46 ^o^ | -0.0377 | 0.0021 | -17.71 | <2e-16 *** | 12807 |
|  | Latitude > 46 ^o^ | -0.0260 | 0.0017 | -15.48 | <2e-16 *** |  |
|  | Intercept | -1.7150 | 0.2164 | -12.69 | 2.31e-15 *** |  |
| **Metabolic rate** | Mass specific metabolic rate | 0.0162 | 0.0006 | 26.22 | <2e-16 *** | 12479 |
|  | Intercept | -3.3080 | 0.1963 | -16.85 | <2e-16 *** |  |
| Generation time | Body size | -0.0008 | 0.0003 | -2.32 | 0.0201 * | 13120 |
|  | Intercept | -3.0522 | 0.2075 | -20.48 | <2e-16 *** |  |
| Temperature | Temperature | 0.0209 | 0.0020 | 10.13 | <2e-16 *** | 13016 |
|  | Intercept | -3.3545 | 0.1997 | -16.80 | <2e-16 *** |  |
| **Best complex model** | Midpoint latitude | -0.0124 | 0.0027 | -4.66 | 3.21e-06 *** | 12279 |
|  | Body size | 0.0038 | 0.0003 | 11.12 | <2e-16 *** |  |
|  | Temperature | -0.0393 | 0.0038 | -10.30 | <2e-16 *** |  |
|  | Mass specific metabolic rate | 0.0246 | 0.0009 | 27.94 | <2e-16 *** |  |
|  | Intercept | -2.6029 | 0.2411 | -10.79 | <2e-16 *** |  |
