# Supplementary material for: Metabolic Rate and Climatic Fluctuations Shape Continental Wide Pattern of Genetic Divergence and Biodiversity in Fishes
Source: PLoS One. 2013 Jul 29;8(7):e70296. doi: 10.1371/journal.pone.0070296 (PMC3726496; doi:10.1371/journal.pone.0070296)
Supplement: Table S2 — Pearson correlations between explanatory variables. (DOCX) [file pone.0070296.s003.docx]

**Supplementary Table**

**Table S2.** Pearson correlations between explanatory variables.

|  | **Midpoint latitude** | **Body size** | **Temperature** | **Mass specific metabolic rate** |
| --- | --- | --- | --- | --- |
| **Midpoint latitude** | - |  |  |  |
| **Body size** | 0.110 | - |  |  |
| **Temperature** | -0.706 | 0.228 | - |  |
| **Mass specific metabolic rate** | 0.094 | -0.478 | 0.565 | - |
