# Supplementary material for: Metabolic Rate and Climatic Fluctuations Shape Continental Wide Pattern of Genetic Divergence and Biodiversity in Fishes
Source: PLoS One. 2013 Jul 29;8(7):e70296. doi: 10.1371/journal.pone.0070296 (PMC3726496; doi:10.1371/journal.pone.0070296)
Supplement: Text S1 — List of species included in the intraspecific divergence analyses. (DOCX) [file pone.0070296.s004.docx]

**Supplementary text**

**Text S1.** List of species included in the intraspecific divergence analyses.

*Acantharchus pomotis, Acrocheilus alutaceus, Adinia xenica, Agosia chrysogaster, Ambloplites ariommus, Ambloplites constellatus, Ambloplites rupestris, Ameiurus brunneus, Ameiurus melas, Ameiurus natalis, Ameiurus nebulosus, Ameiurus platycephalus, Ameiurus serracanthus, Ammocrypta beanii, Ammocrypta bifascia, Ammocrypta clara, Ammocrypta meridiana, Ammocrypta pellucida, Ammocrypta vivax, Archoplites interruptus, Campostoma anomalum, Campostoma oligolepis, Campostoma ornatum, Campostoma pauciradii, Carpiodes carpio, Carpiodes cyprinus, Carpiodes velifer, Catostomus ardens, Catostomus bernardini, Catostomus catostomus, Catostomus clarkii, Catostomus columbianus, Catostomus commersonii, Catostomus insignis, Catostomus macrocheilus, Catostomus microps, Catostomus occidentalis, Catostomus platyrhynchus, Catostomus plebeius, Catostomus santaanae, Centrarchus macropterus, Clinostomus elongatus, Clinostomus funduloides, Coregonus artedi, Coregonus autumnalis, Coregonus clupeaformis, Coregonus hoyi, Coregonus laurettae, Coregonus nasus, Coregonus nigripinnis, Coregonus sardinella, Coregonus zenithicus, Cottus aleuticus, Cottus asper, Cottus bairdii, Cottus beldingii, Cottus caeruleomentum, Cottus carolinae, Cottus chattahoochee, Cottus cognatus, Cottus girardi, Cottus gulosus, Cottus hypselurus, Cottus klamathensis, Cottus perplexus, Cottus pitensis, Cottus poecilopus, Cottus rhotheus, Cottus ricei, Cottus tallapoosae, Cottus tenuis, Couesius plumbeus, Crystallaria asprella, Cycleptus elongatus, Cyprinella analostana, Cyprinella caerulea, Cyprinella callisema, Cyprinella callistia, Cyprinella camura, Cyprinella chloristia, Cyprinella galactura, Cyprinella gibbsi, Cyprinella leedsi, Cyprinella nivea, Cyprinella proserpina, Cyprinella pyrrhomelas, Cyprinella spiloptera, Cyprinella trichroistia, Cyprinella venusta, Cyprinella whipplei, Cyprinodon variegatus, Dallia pectoralis, Dionda episcopa, Elassoma alabamae, Elassoma evergladei, Elassoma zonatum, Enneacanthus chaetodon, Enneacanthus gloriosus, Enneacanthus obesus, Erimonax monachus, Erimystax cahni, Erimystax dissimilis, Erimystax harryi, Erimystax insignis, Erimystax x-punctatus, Erimyzon oblongus, Erimyzon sucetta, Erimyzon tenuis, Esox americanus, Esox lucius, Esox masquinongy, Esox niger, Etheostoma acuticeps, Etheostoma akatulo, Etheostoma aquali, Etheostoma artesiae, Etheostoma asprigene, Etheostoma atripinne, Etheostoma baileyi, Etheostoma barbouri, Etheostoma barrenense, Etheostoma basilare, Etheostoma bellator, Etheostoma bellum, Etheostoma bison, Etheostoma blennioides, Etheostoma blennius, Etheostoma boschungi, Etheostoma brevirostrum, Etheostoma burri, Etheostoma caeruleum, Etheostoma camurum, Etheostoma cervus, Etheostoma chermocki, Etheostoma chienense, Etheostoma chlorobranchium, Etheostoma chlorosomum, Etheostoma cinereum, Etheostoma collettei, Etheostoma collis, Etheostoma coosae, Etheostoma corona, Etheostoma cragini, Etheostoma crossopterum, Etheostoma davisoni, Etheostoma derivativum, Etheostoma ditrema, Etheostoma douglasi, Etheostoma duryi, Etheostoma edwini, Etheostoma etnieri, Etheostoma etowahae, Etheostoma exile, Etheostoma flabellare, Etheostoma flavum, Etheostoma fonticola, Etheostoma forbesi, Etheostoma fragi, Etheostoma fricksium, Etheostoma fusiforme, Etheostoma gracile, Etheostoma grahami, Etheostoma gutselli, Etheostoma histrio, Etheostoma inscriptum, Etheostoma jessiae, Etheostoma jordani, Etheostoma juliae, Etheostoma kanawhae, Etheostoma kantuckeense, Etheostoma kennicotti, Etheostoma lachneri, Etheostoma lawrencei, Etheostoma lepidum, Etheostoma longimanum, Etheostoma luteovinctum, Etheostoma lynceum, Etheostoma maculatum, Etheostoma meadiae, Etheostoma microlepidum, Etheostoma microperca, Etheostoma moorei, Etheostoma neopterum, Etheostoma nigripinne, Etheostoma nigrum, Etheostoma nuchale, Etheostoma obeyense, Etheostoma okaloosae, Etheostoma olivaceum, Etheostoma olmstedi, Etheostoma oophylax, Etheostoma osburni, Etheostoma parvipinne, Etheostoma percnurum, Etheostoma perlongum, Etheostoma phytophilum, Etheostoma podostemone, Etheostoma pottsii, Etheostoma proeliare, Etheostoma pseudovulatum, Etheostoma punctulatum, Etheostoma pyrrhogaster, Etheostoma radiosum, Etheostoma rafinesquei, Etheostoma ramseyi, Etheostoma raneyi, Etheostoma rubrum, Etheostoma rufilineatum, Etheostoma rupestre, Etheostoma sagitta, Etheostoma saludae, Etheostoma sanguifluum, Etheostoma serrifer, Etheostoma simoterum, Etheostoma sitikuense, Etheostoma smithi, Etheostoma spectabile, Etheostoma squamiceps, Etheostoma stigmaeum, Etheostoma striatulum, Etheostoma susanae, Etheostoma swaini, Etheostoma swannanoa, Etheostoma tallapoosae, Etheostoma tecumsehi, Etheostoma tetrazonum, Etheostoma thalassinum, Etheostoma tippecanoe, Etheostoma tuscumbia, Etheostoma uniporum, Etheostoma variatum, Etheostoma virgatum, Etheostoma vitreum, Etheostoma vulneratum, Etheostoma wapiti, Etheostoma whipplei, Etheostoma zonale, Etheostoma zonifer, Etheostoma zonistium, Exoglossum laurae, Exoglossum maxillingua, Fundulus blairae, Fundulus catenatus, Fundulus chrysotus, Fundulus cingulatus, Fundulus diaphanus, Fundulus dispar, Fundulus escambiae, Fundulus euryzonus, Fundulus heteroclitus, Fundulus jenkinsi, Fundulus kansae, Fundulus lima, Fundulus lineolatus, Fundulus notatus, Fundulus olivaceus, Fundulus rubrifrons, Fundulus sciadicus, Fundulus similis, Fundulus stellifer, Fundulus zebrinus, Gambusia affinis, Gambusia holbrooki, Gila coerulea, Gila elegans, Gila nigrescens, Gila orcuttii, Hemitremia flammea, Hesperoleucus symmetricus, Heterandria formosa, Hybognathus argyritis, Hybognathus hankinsoni, Hybognathus nuchalis, Hybognathus placitus, Hybognathus regius, Hybopsis amblops, Hybopsis amnis, Hybopsis hyposinotus, Hybopsis hypsinotus, Hybopsis lineapunctata, Hybopsis rubrifrons, Hybopsis winchelli, Hybopsis zanema, Hypentelium etowanum, Hypentelium nigricans, Ictalurus lupus, Ictalurus punctatus, Ictiobus bubalus, Ictiobus cyprinellus, Ictiobus niger, Jordanella floridae, Lepomis auritus, Lepomis cyanellus, Lepomis gibbosus, Lepomis gulosus, Lepomis humilis, Lepomis macrochirus, Lepomis marginatus, Lepomis megalotis, Lepomis microlophus, Lepomis miniatus, Lepomis punctatus, Lepomis symmetricus, Leptolucania ommata, Lucania goodei, Luxilus albeolus, Luxilus cerasinus, Luxilus chrysocephalus, Luxilus coccogenis, Luxilus cornutus, Luxilus zonistius, Lythrurus alegnotus, Lythrurus ardens, Lythrurus atrapiculus, Lythrurus bellus, Lythrurus fasciolaris, Lythrurus fumeus, Lythrurus lirus, Lythrurus matutinus, Lythrurus roseipinnis, Lythrurus snelsoni, Lythrurus umbratilis, Macrhybopsis aestivalis, Macrhybopsis gelida, Macrhybopsis hyostoma, Macrhybopsis marconis, Macrhybopsis meeki, Macrhybopsis storeriana, Margariscus margarita, Meda fulgida, Micropterus cataractae, Micropterus coosae, Micropterus dolomieu, Micropterus floridanus, Micropterus notius, Micropterus punctulatus, Micropterus salmoides, Micropterus treculii, Minytrema melanops, Moapa coriacea, Morone chrysops, Morone mississippiensis, Moxostoma anisurum, Moxostoma ariommum, Moxostoma breviceps, Moxostoma carinatum, Moxostoma congestum, Moxostoma duquesnii, Moxostoma erythrurum, Moxostoma hubbsi, Moxostoma lachneri, Moxostoma macrolepidotum, Moxostoma pappillosum, Moxostoma poecilurum, Moxostoma robustum, Moxostoma rupiscartes, Moxostoma valenciennesi, Mylocheilus caurinus, Myoxocephalus thompsonii, Nocomis asper, Nocomis biguttatus, Nocomis effusus, Nocomis leptocephalus, Nocomis micropogon, Nocomis platyrhynchus, Nocomis raneyi, Notemigonus crysoleucas, Notropis altipinnis, Notropis amabilis, Notropis ammophilus, Notropis amoenus, Notropis amplamala, Notropis anogenus, Notropis ariommus, Notropis asperifrons, Notropis atherinoides, Notropis atrocaudalis, Notropis baileyi, Notropis bairdi, Notropis bifrenatus, Notropis blennius, Notropis boops, Notropis buccatus, Notropis buchanani, Notropis cahabae, Notropis chalybaeus, Notropis chihuahua, Notropis chiliticus, Notropis chlorocephalus, Notropis chrosomus, Notropis cummingsae, Notropis dorsalis, Notropis edwardraneyi, Notropis girardi, Notropis harperi, Notropis heterodon, Notropis heterolepis, Notropis hudsonius, Notropis hypsilepis, Notropis jemezanus, Notropis leuciodus, Notropis longirostris, Notropis lutipinnis, Notropis maculatus, Notropis mekistocholas, Notropis micropteryx, Notropis nubilus, Notropis ortenburgeri, Notropis ozarcanus, Notropis percobromus, Notropis perpallidus, Notropis petersoni, Notropis photogenis, Notropis procne, Notropis rubellus, Notropis rubricroceus, Notropis rupestris, Notropis sabinae, Notropis scabriceps, Notropis scepticus, Notropis semperasper, Notropis shumardi, Notropis simus, Notropis spectrunculus, Notropis stilbius, Notropis stramineus, Notropis telescopus, Notropis texanus, Notropis topeka, Notropis uranoscopus, Notropis volucellus, Notropis xaenocephalus, Noturus albater, Noturus crypticus, Noturus elegans, Noturus eleutherus, Noturus exilis, Noturus flavipinnis, Noturus flavus, Noturus furiosus, Noturus gyrinus, Noturus hildebrandi, Noturus insignis, Noturus lachneri, Noturus leptacanthus, Noturus miurus, Noturus nocturnus, Noturus phaeus, Noturus stigmosus, Noturus taylori, Novumbra hubbsi, Opsopoeodus emiliae, Oregonichthys crameri, Oregonichthys kalawatseti, Perca flavescens, Percina aurantiaca, Percina aurolineata, Percina aurora, Percina austroperca, Percina burtoni, Percina caprodes, Percina carbonaria, Percina copelandi, Percina crassa, Percina cymatotaenia, Percina evides, Percina fulvitaenia, Percina gymnocephala, Percina jenkinsi, Percina kathae, Percina lenticula, Percina macrocephala, Percina macrolepida, Percina maculata, Percina nasuta, Percina nebulosa, Percina nevisense, Percina nigrofasciata, Percina notogramma, Percina oxyrhynchus, Percina palmaris, Percina peltata, Percina phoxocephala, Percina rex, Percina roanoka, Percina sciera, Percina shumardi, Percina smithvanizi, Percina squamata, Percina uranidea, Percina vigil, Phenacobius crassilabrum, Phenacobius mirabilis, Phenacobius teretulus, Phenacobius uranops, Phoxinus eos, Phoxinus erythrogaster, Phoxinus neogaeus, Phoxinus oreas, Phoxinus saylori, Pimephales notatus, Pimephales promelas, Pimephales tenellus, Pimephales vigilax, Plagopterus argentissimus, Platygobio gracilis, Poecilia latipinna, Poeciliopsis occidentalis, Pomoxis annularis, Pomoxis nigromaculatus, Prosopium coulterii, Prosopium cylindraceum, Prosopium williamsoni, Pteronotropis euryzonus, Pteronotropis grandipinnis, Pteronotropis hubbsi, Pteronotropis hypselopterus, Pteronotropis metallicus,Pteronotropis signipinnis, Pteronotropis welaka, Ptychocheilus grandis, Ptychocheilus lucius, Ptychocheilus oregonensis, Ptychocheilus umpquae, Pylodictis olivaris, Rhinichthys atratulus, Rhinichthys cataractae, Rhinichthys cobitis, Rhinichthys evermanni, Rhinichthys falcatus, Rhinichthys obtusus, Rhinichthys osculus, Rhinichthys umatilla, Richardsonius balteatus, Salvelinus alpinus, Salvelinus confluentus, Salvelinus fontinalis, Salvelinus malma, Salvelinus namaycush, Sander canadensis, Sander vitreus, Semotilus atromaculatus, Semotilus corporalis, Semotilus thoreauianus, Stenodus leucichthys, Thoburnia rhothoeca, Thymallus arcticus, Umbra limi, Umbra pygmaea, Xyrauchen texanus.*
