# Supplementary material for: Metabolic Rate and Climatic Fluctuations Shape Continental Wide Pattern of Genetic Divergence and Biodiversity in Fishes
Source: PLoS One. 2013 Jul 29;8(7):e70296. doi: 10.1371/journal.pone.0070296 (PMC3726496; doi:10.1371/journal.pone.0070296)
Supplement: Text S2 — List of species included in the interspecific divergence analyses. (DOCX) [file pone.0070296.s005.docx]

**Supplementary text**

**Text S2.** List of species included in the interspecific divergence analyses.

*Acrocheilus alutaceus, Adinia xenica, Ambloplites ariommus, Ambloplites cavifrons, Ambloplites constellatus, Ambloplites rupestris, Ameiurus brunneus, Ameiurus melas, Ameiurus natalis, Ameiurus nebulosus, Ameiurus platycephalus, Ameiurus serracanthus, Ammocrypta clara, Ammocrypta meridiana, Ammocrypta pellucida, Ammocrypta vivax, Archoplites interruptus, Astyanax mexicanus, Atractosteus spatula, Campostoma oligolepis, Campostoma ornatum, Campostoma pauciradii, Catostomus ardens, Catostomus bernardini, Catostomus catostomus, Catostomus clarkii, Catostomus columbianus, Catostomus commersonii, Catostomus insignis, Catostomus macrocheilus, Catostomus platyrhynchus, Catostomus plebeius, Catostomus santaanae, Catostomus tahoensis, Chrosomus cumberlandensis, Clinostomus elongatus, Clinostomus funduloides, Coregonus clupeaformis, Coregonus huntsmani, Coregonus nasus, Coregonus sardinella, Cottus aleuticus, Cottus asper, Cottus beldingii, Cottus carolinae, Cottus girardi, Cottus gulosus, Cottus hypselurus, Cottus klamathensis, Cottus leiopomus, Cottus perplexus, Cottus pitensis, Cottus poecilopus, Cottus rhotheus, Cottus ricei, Cottus tenuis, Couesius plumbeus, Crystallaria asprella, Cycleptus elongatus, Cyprinella analostana, Cyprinella caerulea, Cyprinella callistia, Cyprinella camura, Cyprinella chloristia, Cyprinella galactura, Cyprinella gibbsi, Cyprinella leedsi, Cyprinella nivea, Cyprinella proserpina, Cyprinella pyrrhomelas, Cyprinella spiloptera, Cyprinella trichroistia, Cyprinella venusta, Cyprinella whipplei, Cyprinella xaenura, Elassoma alabamae, Elassoma evergladei, Elassoma okefenokee, Elassoma zonatum, Enneacanthus chaetodon, Enneacanthus gloriosus, Enneacanthus obesus, Erimonax monachus, Erimystax cahni, Erimystax dissimilis, Erimystax harryi, Erimystax insignis, Erimystax x-punctatus, Erimyzon oblongus, Erimyzon sucetta, Erimyzon tenuis, Etheostoma acuticeps, Etheostoma akatulo, Etheostoma artesiae, Etheostoma asprigene, Etheostoma atripinne, Etheostoma australe, Etheostoma baileyi, Etheostoma barbouri, Etheostoma barrenense, Etheostoma basilare, Etheostoma bellator, Etheostoma bellum, Etheostoma bison, Etheostoma blennioides, Etheostoma blennius, Etheostoma boschungi, Etheostoma brevirostrum, Etheostoma burri, Etheostoma chermocki, Etheostoma chienense, Etheostoma chlorobranchium, Etheostoma chlorosomum, Etheostoma chuckwachatte, Etheostoma collettei, Etheostoma collis, Etheostoma colorosum, Etheostoma coosae, Etheostoma corona, Etheostoma cragini, Etheostoma crossopterum, Etheostoma davisoni, Etheostoma denoncourti, Etheostoma derivativum, Etheostoma ditrema, Etheostoma douglasi, Etheostoma duryi, Etheostoma edwini, Etheostoma etnieri, Etheostoma etowahae, Etheostoma euzonum, Etheostoma exile, Etheostoma flabellare, Etheostoma flavum, Etheostoma fonticola, Etheostoma forbesi, Etheostoma fragi, Etheostoma fricksium, Etheostoma fusiforme, Etheostoma gracile, Etheostoma grahami, Etheostoma gutselli, Etheostoma histrio, Etheostoma inscriptum, Etheostoma jessiae, Etheostoma jordani, Etheostoma juliae, Etheostoma kanawhae, Etheostoma kantuckeense, Etheostoma kennicotti, Etheostoma lachneri, Etheostoma lawrencei, Etheostoma lepidum, Etheostoma longimanum, Etheostoma luteovinctum, Etheostoma lynceum, Etheostoma maculatum, Etheostoma meadiae, Etheostoma microlepidum, Etheostoma microperca, Etheostoma moorei, Etheostoma neopterum, Etheostoma nigripinne, Etheostoma nigrum, Etheostoma nuchale, Etheostoma obeyense, Etheostoma okaloosae, Etheostoma olivaceum, Etheostoma olmstedi, Etheostoma oophylax, Etheostoma osburni, Etheostoma pallididorsum, Etheostoma parvipinne, Etheostoma perlongum, Etheostoma phytophilum, Etheostoma podostemone, Etheostoma pottsii, Etheostoma proeliare, Etheostoma pseudovulatum, Etheostoma punctulatum, Etheostoma radiosum, Etheostoma rafinesquei, Etheostoma ramseyi, Etheostoma raneyi, Etheostoma rubrum, Etheostoma rufilineatum, Etheostoma rupestre, Etheostoma sagitta, Etheostoma saludae, Etheostoma sanguifluum, Etheostoma serrifer, Etheostoma smithi, Etheostoma spectabile, Etheostoma squamiceps, Etheostoma stigmaeum, Etheostoma striatulum, Etheostoma susanae, Etheostoma swaini, Etheostoma swannanoa, Etheostoma tallapoosae, Etheostoma tecumsehi, Etheostoma tetrazonum, Etheostoma thalassinum, Etheostoma tippecanoe, Etheostoma trisella, Etheostoma tuscumbia, Etheostoma variatum, Etheostoma virgatum, Etheostoma vitreum, Etheostoma vulneratum, Etheostoma whipplei, Etheostoma zonale, Etheostoma zonifer, Etheostoma zonistium, Exoglossum laurae, Exoglossum maxillingua, Fundulus blairae, Fundulus catenatus, Fundulus chrysotus, Fundulus cingulatus, Fundulus dispar, Fundulus escambiae, Fundulus euryzonus, Fundulus heteroclitus, Fundulus kansae, Fundulus lineolatus, Fundulus notatus, Fundulus olivaceus, Fundulus rubrifrons, Fundulus sciadicus, Fundulus similis, Fundulus stellifer, Fundulus zebrinus, Gambusia affinis, Gambusia holbrooki, Gila coerulea, Gila elegans, Gila nigrescens, Gila orcuttii, Gila pandora, Gila robusta, Hesperoleucus symmetricus, Hybognathus argyritis, Hybognathus hankinsoni, Hybognathus nuchalis, Hybognathus placitus, Hybognathus regius, Hybopsis amblops, Hybopsis amnis, Hybopsis hyposinotus, Hybopsis hypsinotus, Hybopsis lineapunctata, Hybopsis rubrifrons, Hybopsis winchelli, Hybopsis zanema, Hypentelium etowanum, Hypentelium nigricans, Hypentelium roanokense, Ictalurus lupus, Ictalurus punctatus, Lepisosteus oculatus, Lepisosteus osseus, Lepisosteus platostomus, Lepisosteus platyrhincus, Lepomis auritus, Lepomis gibbosus, Lepomis gulosus, Lepomis humilis, Lepomis macrochirus, Lepomis microlophus, Lepomis miniatus, Lepomis punctatus, Lucania goodei, Lucania parva, Luxilus albeolus, Luxilus cerasinus, Luxilus chrysocephalus, Luxilus coccogenis, Luxilus cornutus, Luxilus zonatus, Luxilus zonistius, Lythrurus alegnotus, Lythrurus ardens, Lythrurus atrapiculus, Lythrurus bellus, Lythrurus fasciolaris, Lythrurus lirus, Lythrurus matutinus, Lythrurus snelsoni, Lythrurus umbratilis, Macrhybopsis aestivalis, Macrhybopsis gelida, Macrhybopsis hyostoma, Macrhybopsis marconis, Macrhybopsis meeki, Macrhybopsis storeriana, Margariscus margarita, Micropterus cataractae, Micropterus coosae, Micropterus dolomieu, Micropterus floridanus, Micropterus notius, Micropterus salmoides, Minytrema melanops, Moxostoma anisurum, Moxostoma ariommum, Moxostoma austrinum, Moxostoma breviceps, Moxostoma carinatum, Moxostoma cervinum, Moxostoma congestum, Moxostoma duquesnii, Moxostoma erythrurum, Moxostoma hubbsi, Moxostoma lachneri, Moxostoma pappillosum, Moxostoma poecilurum, Moxostoma robustum, Moxostoma rupiscartes, Moxostoma valenciennesi, Mylocheilus caurinus, Myoxocephalus quadricornis, Myoxocephalus thompsonii, Nocomis asper, Nocomis biguttatus, Nocomis effusus, Nocomis leptocephalus, Nocomis micropogon, Nocomis platyrhynchus, Nocomis raneyi, Notropis altipinnis, Notropis ammophilus, Notropis amoenus, Notropis amplamala, Notropis anogenus, Notropis ariommus, Notropis asperifrons, Notropis atherinoides, Notropis atrocaudalis, Notropis baileyi, Notropis bairdi, Notropis bifrenatus, Notropis blennius, Notropis boops, Notropis buccatus, Notropis cahabae, Notropis chalybaeus, Notropis chihuahua, Notropis chiliticus, Notropis chlorocephalus, Notropis chrosomus, Notropis cummingsae, Notropis dorsalis, Notropis edwardraneyi, Notropis girardi, Notropis greenei, Notropis harperi, Notropis heterodon, Notropis heterolepis, Notropis hudsonius, Notropis hypsilepis, Notropis leuciodus, Notropis longirostris, Notropis lutipinnis, Notropis maculatus, Notropis mekistocholas, Notropis micropteryx, Notropis nubilus, Notropis ortenburgeri, Notropis ozarcanus, Notropis percobromus, Notropis perpallidus, Notropis petersoni, Notropis photogenis, Notropis potteri, Notropis procne, Notropis rubellus, Notropis rubricroceus, Notropis rupestris, Notropis sabinae, Notropis scabriceps, Notropis scepticus, Notropis semperasper, Notropis shumardi, Notropis simus pecoensis, Notropis spectrunculus, Notropis stilbius, Notropis stramineus, Notropis telescopus, Notropis texanus, Notropis topeka, Notropis uranoscopus, Notropis wickliffi, Notropis xaenocephalus, Noturus albater, Noturus crypticus, Noturus elegans, Noturus eleutherus, Noturus exilis, Noturus flavater, Noturus flavipinnis, Noturus flavus, Noturus funebris, Noturus furiosus, Noturus gyrinus, Noturus hildebrandi, Noturus insignis, Noturus lachneri, Noturus leptacanthus, Noturus miurus, Noturus nocturnus, Noturus phaeus, Noturus stanauli, Noturus stigmosus, Noturus taylori, Opsopoeodus emiliae, Oregonichthys crameri, Oregonichthys kalawatseti, Perca flavescens, Percina antesella, Percina aurantiaca, Percina aurolineata, Percina aurora, Percina austroperca, Percina brevicauda, Percina burtoni, Percina carbonaria, Percina copelandi, Percina crassa, Percina cymatotaenia, Percina evides, Percina gymnocephala, Percina jenkinsi, Percina kathae, Percina lenticula, Percina macrocephala, Percina macrolepida, Percina maculata, Percina nasuta, Percina nebulosa, Percina nevisense, Percina nigrofasciata, Percina notogramma, Percina oxyrhynchus, Percina palmaris, Percina pantherina, Percina peltata, Percina phoxocephala, Percina rex, Percina roanoka, Percina sciera, Percina shumardi, Percina smithvanizi, Percina squamata, Percina vigil, Phenacobius crassilabrum, Phenacobius mirabilis, Phenacobius teretulus, Phenacobius uranops, Phoxinus eos, Phoxinus erythrogaster, Phoxinus neogaeus, Phoxinus oreas, Phoxinus saylori, Pimephales notatus, Pimephales promelas, Pimephales tenellus, Pimephales vigilax, Platygobio gracilis, Poecilia latipinna, Poecilia mexicana, Pomoxis annularis, Pomoxis nigromaculatus, Prosopium coulterii, Prosopium cylindraceum, Prosopium williamsoni, Pteronotropis euryzonus, Pteronotropis grandipinnis, Pteronotropis hubbsi, Pteronotropis hypselopterus, Pteronotropis metallicus, Pteronotropis signipinnis, Pteronotropis welaka, Ptychocheilus grandis, Ptychocheilus lucius, Ptychocheilus oregonensis, Ptychocheilus umpquae, Pylodictis olivaris, Rhinichthys atratulus, Rhinichthys cobitis, Rhinichthys evermanni, Rhinichthys falcatus, Rhinichthys obtusus, Rhinichthys osculus, Rhinichthys umatilla, Richardsonius balteatus, Salvelinus alpinus, Salvelinus confluentus, Salvelinus fontinalis, Salvelinus malma, Salvelinus namaycush, Sander canadensis, Sander vitreus, Semotilus atromaculatus, Semotilus corporalis, Semotilus lumbee, Semotilus thoreauianus, Stenodus leucichthys, Thoburnia rhothoeca, Xyrauchen texanus.*
