# Supplementary material for: Metabolic Rate and Climatic Fluctuations Shape Continental Wide Pattern of Genetic Divergence and Biodiversity in Fishes
Source: PLoS One. 2013 Jul 29;8(7):e70296. doi: 10.1371/journal.pone.0070296 (PMC3726496; doi:10.1371/journal.pone.0070296)
Supplement: Text S3 — List of species included in the analyses testing the correlation between mass specific metabolic rate and mutation rate. (DOCX) [file pone.0070296.s006.docx]

**Supplementary text**

**Text S3.** List of species included in the analyses testing the correlation between mass specific metabolic rate and mutation rate.

*Ambloplites ariommus, Ambloplites cavifrons, Ambloplites constellatus, Ambloplites rupestris, Ameiurus melas, Ameiurus nebulosus, Ammocrypta clara, Ammocrypta meridiana, Ammocrypta pellucida, Ammocrypta vivax, Catostomus ardens, Catostomus clarkii, Catostomus macrocheilus, Catostomus santaanae, Chrosomus cumberlandensis, Clinostomus elongatus, Clinostomus funduloides, Coregonus clupeaformis, Coregonus nasus, Cottus asper, Cottus beldingii, Cottus gulosus, Cottus klamathensis, Cottus leiopomus, Cottus tenuis, Couesius plumbeus, Cyprinella analostana, Cyprinella camura, Cyprinella chloristia, Cyprinella galactura, Cyprinella gibbsi, Cyprinella spiloptera, Cyprinella trichroistia, Cyprinella whipplei, Elassoma alabamae, Elassoma evergladei, Elassoma okefenokee, Elassoma zonatum, Enneacanthus gloriosus, Enneacanthus obesus, Erimystax cahni, Erimystax dissimilis, Erimystax harryi, Erimystax x-punctatus, Erimyzon oblongus, Erimyzon sucetta, Etheostoma akatulo, Etheostoma barrenense, Etheostoma bellator, Etheostoma blennioides, Etheostoma blennius, Etheostoma boschungi, Etheostoma chienense, Etheostoma collis, Etheostoma crossopterum, Etheostoma denoncourti, Etheostoma duryi, Etheostoma edwini, Etheostoma etowahae, Etheostoma euzonum, Etheostoma flavum, Etheostoma fonticola, Etheostoma fricksium, Etheostoma gracile, Etheostoma grahami, Etheostoma gutselli, Etheostoma inscriptum, Etheostoma jessiae, Etheostoma jordani, Etheostoma kanawhae, Etheostoma lachneri, Etheostoma longimanum, Etheostoma lynceum, Etheostoma meadiae, Etheostoma microlepidum, Etheostoma moorei, Etheostoma neopterum, Etheostoma nigripinne, Etheostoma nigrum, Etheostoma olivaceum, Etheostoma olmstedi, Etheostoma oophylax, Etheostoma osburni, Etheostoma parvipinne, Etheostoma phytophilum, Etheostoma podostemone, Etheostoma pottsii, Etheostoma proeliare, Etheostoma rafinesquei, Etheostoma ramseyi, Etheostoma rubrum, Etheostoma rupestre, Etheostoma saludae, Etheostoma sanguifluum, Etheostoma smithi, Etheostoma stigmaeum, Etheostoma striatulum, Etheostoma swannanoa, Etheostoma tallapoosae, Etheostoma tetrazonum, Etheostoma tippecanoe, Etheostoma tuscumbia, Etheostoma zonale, Etheostoma zonifer, Exoglossum laurae, Exoglossum maxillingua, Forbesichthys agassizii, Fundulus blairae, Fundulus catenatus, Fundulus cingulatus, Fundulus dispar, Fundulus escambiae, Fundulus euryzonus, Fundulus kansae, Fundulus lineolatus, Fundulus olivaceus, Fundulus rubrifrons, Fundulus stellifer, Fundulus zebrinus, Gambusia affinis, Gambusia holbrooki, Gila nigrescens, Gila pandora, Hybognathus argyritis, Hybognathus hankinsoni, Hybognathus nuchalis, Hybognathus regius, Hybopsis amblops, Hybopsis winchelli, Hypentelium nigricans, Hypentelium roanokense, Ictalurus lupus, Ictalurus punctatus, Lepisosteus oculatus, Lepisosteus osseus, Lepisosteus platostomus, Lepisosteus platyrhincus, Lepomis humilis, Lepomis macrochirus, Lepomis miniatus, Lepomis punctatus, Lucania goodei, Lucania parva, Luxilus albeolus, Luxilus cornutus, Lythrurus alegnotus, Lythrurus ardens, Lythrurus bellus, Lythrurus fasciolaris, Lythrurus matutinus, Lythrurus umbratilis, Macrhybopsis aestivalis, Macrhybopsis gelida, Macrhybopsis storeriana, Margariscus margarita, Micropterus floridanus, Micropterus salmoides, Moxostoma austrinum, Moxostoma breviceps, Moxostoma congestum, Moxostoma duquesnii, Moxostoma hubbsi, Moxostoma poecilurum, Nocomis biguttatus, Nocomis effusus, Nocomis micropogon, Nocomis raneyi, Notropis ammophilus, Notropis atherinoides, Notropis bairdi, Notropis blennius, Notropis boops, Notropis chalybaeus, Notropis chlorocephalus, Notropis chrosomus, Notropis heterolepis, Notropis hypsilepis, Notropis longirostris, Notropis lutipinnis, Notropis mekistocholas, Notropis micropteryx, Notropis nubilus, Notropis petersoni, Notropis potteri, Notropis rubellus, Notropis rubricroceus, Notropis rupestris, Notropis scepticus, Notropis semperasper, Notropis stilbius, Notropis texanus, Notropis uranoscopus, Notropis xaenocephalus, Noturus eleutherus, Noturus exilis, Noturus flavater, Noturus furiosus, Noturus gyrinus, Noturus insignis, Noturus lachneri, Noturus stigmosus, Oregonichthys crameri, Oregonichthys kalawatseti, Percina aurora, Percina brevicauda, Percina kathae, Percina nasuta, Percina nebulosa, Percina nevisense, Percina oxyrhynchus, Percina palmaris, Percina peltata, Percina phoxocephala, Percina shumardi, Percina smithvanizi, Percina squamata, Percina vigil, Phenacobius crassilabrum, Phenacobius mirabilis, Phenacobius teretulus, Phenacobius uranops, Phoxinus eos, Phoxinus erythrogaster, Phoxinus saylori, Pimephales notatus, Pimephales tenellus, Platygobio gracilis, Prosopium cylindraceum, Prosopium williamsoni, Pteronotropis grandipinnis, Pteronotropis metallicus, Ptychocheilus oregonensis, Ptychocheilus umpquae, Rhinichthys atratulus, Rhinichthys falcatus, Rhinichthys obtusus, Rhinichthys umatilla, Salvelinus alpinus, Salvelinus confluentus, Sander canadensis, Sander vitreus, Semotilus atromaculatus, Semotilus thoreauianus, Typhlichthys subterraneus, Umbra limi, Umbra pygmaea.*
