# Supplementary material for: Metabolic Rate and Climatic Fluctuations Shape Continental Wide Pattern of Genetic Divergence and Biodiversity in Fishes
Source: PLoS One. 2013 Jul 29;8(7):e70296. doi: 10.1371/journal.pone.0070296 (PMC3726496; doi:10.1371/journal.pone.0070296)
Supplement: Text S4 — List of species included in the analyses testing the correlation between mtDNA divergence and nucDNA divergence Data from: Schönhuth S, Mayden RL (2010) Phylogenetic relationships in the genus Cyprinella (Actinopterygii: Cyprinidae) based on mitochondrial and nuclear gene sequences. Mol Phylo Evol 55∶77–98. (DOCX) [file pone.0070296.s007.docx]

**Supplementary text**

**Text S4.** List of species included in the analyses testing the correlation between mtDNA divergence and nucDNA divergence Data from: Schönhuth S & Mayden RL (2010) Phylogenetic relationships in the genus *Cyprinella* (Actinopterygii: Cyprinidae) based on mitochondrial and nuclear gene sequences. Mol Phylo Evol 55: 77-98.

*Campostoma analostoma, Campostoma chloristia, Cyprinella anomalum, Cyprinella camura, Cyprinella galactura, Cyprinella gibbsi, Cyprinella oligolepis, Cyprinella panarcys, Cyprinella paudiradii, Cyprinella procipera, Cyprinella pyrrhomelas, Cyprinella spiloptera, Cyprinella thinlip sp., Cyprinella trichroistia, Cyprinella venusta, Cyprinella xaenura, Cyprinella zenema, Cyprinellapulum, Dionda diaboli, Dionda melanops, Dionda nigrotaeniata, Dionda serena, Hybognathus nuchalis, Hybognathus placitus, Nocomis leptocephalus, Nocomis raneyi, Notropis boucardi, Notropis calientis, Notropis moralesi, Notropis sallaei, Pimephales notatus, Pimephales promelas, Tampichthys catostomops, Tampichthys dichromus, Tampichthys erimyzonops, Tampichthys ipni.*
